# Scarlet Fever

**Published:** 1853-08

**Authors:** M. F. Colby


### SELECTIONS

FROM

### FOREIGN AND HOME MEDICAL JOURNALS.

---

*Scarlet Fever*. By M. F Colby, M. D.—Having just perused, in your Journal of the 16th of February, Dr. Wood's treatment of scarlet fever, I have taken the liberty of sending you my own views and practice in this disease. We country physicians not being so much guarded as others by rules of professional etiquette, are perhaps too apt to adopt independent rules of practice and to adhere to them whether right or wrong. Those who look with contempt on the practice and experience of country physicians, do so without reflection. Our opportunities for observation are far greater than such persons anticipate ; and not being able to visit our patients so often, we are led to anticipate more closely changes which may occur in our absence. We cannot at all times call in a fellow practitioner, to return us a compliment which we may have made him, by strengthening our position with the friends by the declaration that all is right ; but we have to make good our position and abide its consequences—and I can assure you that we are very strictly watched. You see, therefore, that we are often thrown on our own resources, in cases where a city physician could readily call in some one to divide his responsibility.

I have had formerly much experience in scarlet fever. In the year 1832 I had nearly 800 cases, which assumed all shades and all degrees of malignity, from the mildness of the flea-bite to the virulence of the plague. What puzzled me much, was the great diversity in the character of the disease, and the great discrepancy among authors as to its nature and treatment. I found it breaking out here and there without exposure. In some cases several were sick in a family, and in others one or two; and besides, in some cases the rash would appear without the sore throat, in others the affection of the throat without the rash, in others both combined. In some cases in the same family it would manifest itself in enlargement of the parotid gland; but this usually occurred in infants at the breast. In others, the patient would be found in a state of collapse, with mottled, checked or purple appearance beneath or in the skin, without eruption or elevation of the surface. Now, thought I, can this be a contagious disease, exhibiting itself in such a diversity of forms? If so, why does it appear here and there without exposure? And besides, contagious diseases have something specifie in their progress; but in this, all appeared chaotic, not only in its protean character, but also in the treatment as laid down by different authors. Now can order be brought out of confusion? With such reflections I carefully observed the different forms of the disease. At the same time I examined the throats of hundreds within its endemic influence, but who were not sick. In all these cases I found congestion and engorgement of the papillæ of the tongue, with elongation of the soft palate, and some degree of purple appearance about the fauces. The cases of collapse resembled the appearances which I had observed in those exposed to fixed air from burning charcoal, and I had also observed in the latter an eruption somewhat analogous to that of scarlatina.

From these facts I came to the conclusion that the cause producing scarlatina was atmospheric; that the first abnormal change was congestion or passive engorgement, or apoplexy of the mucous membrane, extending from the mouth through all its ramifications in the stomach and intestines, the bronchiæ and air cells of the lungs, as well as in the nostrils and Eustachian tubes to the internal ear. Now what is the first and immediate effect of this congested state of the mucous membrane? An impaired function of the lungs; the blood is imperfectly decarbonized, and so great is the suspension of the function of aeration, that in severe cases the patient falls into a state of collapse, and dies as he would from the respiration of fixed air, without re-action—the dark-mottled appearance of the skin, in these cases, furnishing the only characteristic symptom as pointing out the nature of the disease. In slighter cases this first stage is not observed, and there appears no indication of illness till the eruption appears in the skin. In other more severe cases, the soft palate and fauces become dark and ulcerated, or the retained carbon and perhaps some other noxious principle which induced the disease is spent by irritation of the parotid glands. This engorgement of the capillary vessels, which constitutes essentially the disease (all the other morbid states being the result), produces as stated an impaired state of the function of respiration, or suspension of it. In the latter case the patient falls into a state of collapse, and dies; but when the function is impaired, the carbon is retained and its deleterious effect is manifested by the peculiar eruption which gives the name to this disease. In the congestive stage the vessels of the throat are so over-distended as to lose their vitality, and the parts fall into ulceration or gangrene. If this congestion be removed before the contractile power of the vessels is lost, they fall into a state of collapse; but the function of the mucous surface is not immediately restored—the membrane assuming a shining and glazed appearance. This state of the tongue is without doubt an index to that of the lining membrane of the stomach. By this condition of its mucous coat its function is impaired, and it is from this cause that so many fatal cases of relapse occur from improper food, even after slight cases of scarlatina. I believe that in the mildest cases of this disease, this red and glazed appearance of the tongue is ever an attendant, so much so that an experienced observer can tell by this the character of the disease.

Having taken this view of cause and effect, as constituting this disease in all its protean characteristics, I was led to the adoption of the following practice, and twenty-five years' experience has confirmed my opinion of its correctness. In the early stage while there is still congestion in the mucous membrane, I give an infusion of Cayenne pepper, repeating every ten or fifteen minutes till some effect is observed in the lessening of the purple appearance in the throat—giving it, perhaps, six or eight times. I then give ipecac. to produce free vomiting; and in case the throat is dark and somewhat ulcerated, I add sulphate of copper. In this case the pepper excites the vessels to action, and the concussion produced by the emetic is generally sufficient to relieve the oppressed membrane, and to restore the function of the lungs. If, however, there remain any purple appearance about the fauces and soft palate, I continue the pepper every hour till it is removed. I then treat the disease as mildly inflammatory. As this disease is one of extreme irritability, and the mucous coat of the stomach is in a glazed state, with its function impaired, I give but very little medicine. I usually give mucilages, and occasionally a little warm sage tea, and put the patient under the influence of belladonna. I use the German solution of the extract (three grains to the ounce). This I continue during the whole progress of the disease; and I am confident that no physician who has not given it a thorough trial is aware of its good effect. It lessens the violence of the disease, keeps out the eruption, and effectually equalizes the circulation in the capillary vessels. Since 1832 I have given it as a preventive, with invariable success whenever the directions were complied with. In fact, as I view the disease, no one can take it if brought under the influence of belladonna, for it effectually, by its action on the capillary vessels, pr. vents the congestion which primarily constitutes the disease. The tincture of colchicum and that of the Phytolacca Decandra, would unquestionably exert the same preventive powers. The relapse in scarlatina depends, no doubt, on the retention of too much carbon in the blood, with perhaps some of the deleterious miasm which first induced the disease, and as free action of the skin tends to eliminate this, Dr. Wood's practice would probably prove beneficial. If exposure to cold be avoided, and a careful regimen prescribed for a few days, there will be no danger of relapse. I think no solid food should be taken until the tongue has lost its glazed appearance. Cayenne pepper should never be used after the congestion in the mucous membrane is removed. I must, however, say, that, as far as I have observed the practice of others in this disease, the indiscriminate use of pepper, as practised by Thomsonians, has been far more successful than that of some physicians who treat it as inflammatory from its commencement (without reference to that passive state of engorgement which ushers in the disease), with their calomel, antimony, and cathartics. The one unnecessarily keeps up irritation, but his practice has removed the first morbid condition; while the other permits the congestion to continue, until fatal results ensue, which are often accelerated by calomel and other irritating medicines.

The question may be asked—Do you consider this disease contagious? I answer—Certainly not. In 1832 it prevailed for 50 miles around me, ever occurring in isolated families. Why, then, do those who are exposed take it? They do not take it from the sick, but from the same endemic influence, and you will find its effects in examining all in the same locality. But why do several in the same family have it, while others escape? Because there is similarity of constitution, and the same susceptibility to receive morbid impressions. Such members would have had it if kept a mile apart in the same locality. Such cases I have known. Contagious diseases cannot be prevented by medicine, but belladonna will prevent the access of the disease (if the extract is good); and besides, contagious diseases do not expose to relapses, while exposure to cold or over-eating disposes to it in this, during any period within two weeks.—*Boston Med. and Surg. Journal.*

